# AI-driven fault detection and classification in photovoltaic systems using deep learning techniques

**DOI:** 10.1038/s41598-026-40246-7

**Published:** 2026-03-10

**Authors:** Fatma M. Talaat, Mohamed Salem, Warda M. Shaban

**Affiliations:** 1 Faculty of Artificial Intelligence, Kafrelsheikh University, Kafrelsheikh, 33516 Egypt; 2 Communication and Electronics Engineering Department, Nile Higher Institute for Engineering and Technology, Mansoura, Egypt

**Keywords:** Photovoltaic systems, Fault detection, Renewable energy, AI in solar power, Energy science and technology, Engineering, Mathematics and computing

## Abstract

The growing needs of the world regarding electricity and the exhaustion of fossil fuel resources have aggravated the need to use renewable energy, especially the photovoltaic (PV) systems. Nevertheless, internal defects and external environmental conditions are often known to affect the operational efficiency and reliability of PV modules. This paper presents PVDefectNet, a deep learning-based fault detector and classifier of the PV systems. The proposed solution applies a resnet architecture with data augmentation techniques to enhance its resistance to operating in different operating environments. PVDefectNet is a process based on five stages that include data preparation and preprocessing, model architecture design, training, evaluation and visualization, and performance analysis. The experimental findings indicate that the proposed framework has a high classification performance with an average accuracy of 98, precision of 97.1, recall of 96.5 and F1-score of 96.8 that is better than some of the current methods. Moreover, the visualizations provided by Grad-CAM prove that the model is concentrated on physically significant defect areas, which increases interpretability and reliability. These results suggest that PVDefectNet is a good and clear solution in intelligent monitoring and maintenance of PV systems.

## Introduction

The world’s electrical grid is under increasing pressure from the world’s growing population and wealth. New estimates indicate that by 2050, the yearly global consumption of electricity will have doubled^1^. The current norm for power generation in the industry is the use of fossil fuels and coal, which are traditional energy sources that negatively impact climate change. According to the International Energy Agency^2^, 66% of the world’s carbon dioxide emissions in 2014 came from traditional energy production. Traditional energy sources, however, have their limits as well. In other words, producing electrical energy in a sustainable and ecologically friendly manner can rely heavily on renewable and low-carbon energy sources^3^. According to recent studies, 88% of the world’s electricity will be generated by Photovoltaic (PV) and wind energy systems by the year 2050.

The utilization of electrical and electronic devices has significantly increased over the past decade. The proliferation of electronic devices directly influences the consumption and demand for electrical power^4^. Nevertheless, sources of electric power such as coal, water, and thermal energy are diminishing, and human society cannot rely on them indefinitely. Countries such as China are currently experiencing power shortages and are seeking alternative energy sources, including wind and nuclear power^5^. Solar energy serves as an alternative resource that is abundant and accessible year-round for the long term. PV systems are designed to generate electrical power from solar energy^4,6^.

PV systems are structured with a grid of solar panels, each with its own top panel that absorbs sunlight and is linked to various components and devices that convert it into Direct Current (DC), such as solar cells, diodes, and so on^7^. The inverter-like direct current has been stored in the battery storage after passing through the silicon diode. The inverter takes the DC current and changes it into an Alternate Current (AC), which the other electronics can use. Various parts of the PV system must work together for the system to produce the desired output. Higher power regulation is only possible with careful monitoring and maintenance of the PV system’s individual components^7,8^.

Research on PV energy systems is growing in academia and industry. Increased reliability and complexity have caused PV system automation issues^9^. A fast-growing energy technology is grid-connected PVs. Thus, safe operation and handling are essential. Internal and external issues cause most PV system failures. Internally, wiring or cables are inadequate or broken. External causes include overheating from the room or shade from trees or clouds. Due to surface modifications, solar modules need fault detection. These mistakes and issues affect the module’s output and, worst case, render it useless. Most defects are missed by regular cameras^10^.

**Fig. 1:**
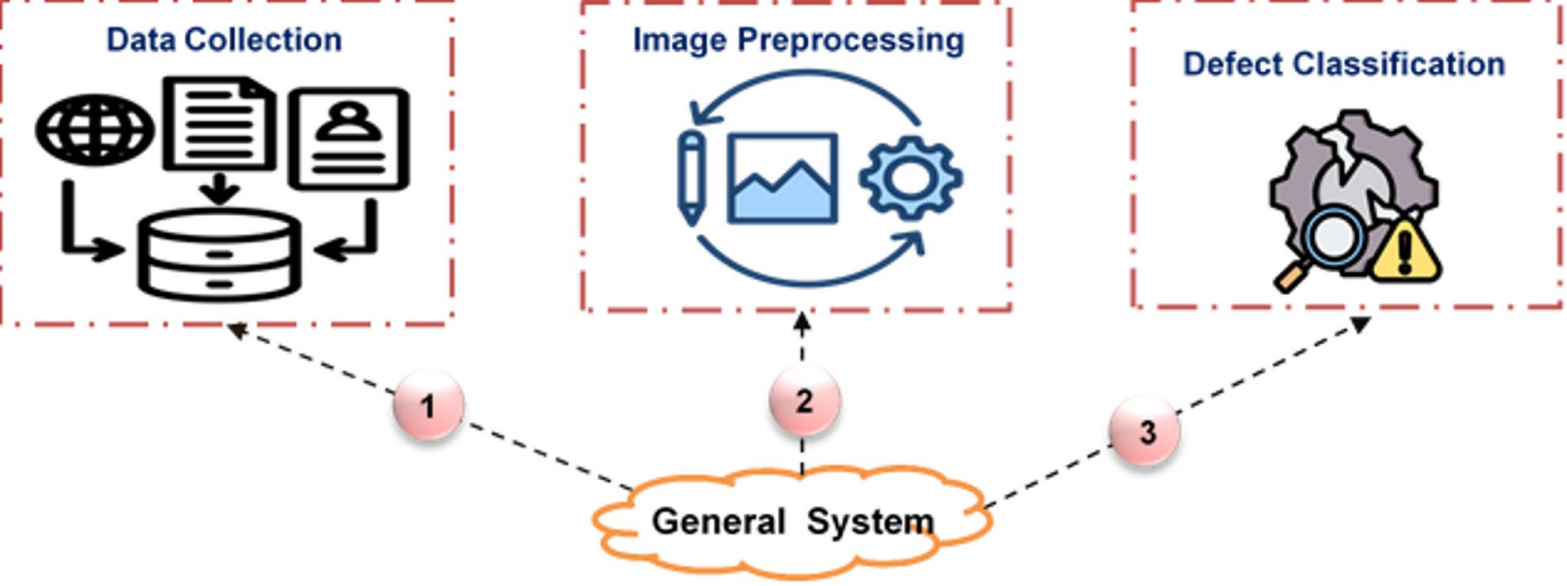
The comprehensive classification system for photovoltaic defects.

Researchers are currently endeavoring to identify solutions to the issues associated with PV system failures^11,12^. A contemporary method for identifying issues involves the utilization of Artificial Intelligence (AI), which has demonstrated capabilities in modeling, controlling, predicting, now casting, diagnosing, and categorizing problems^13–15^. AI is employed at various stages to develop a PV monitoring system. The steps are: (i) data acquisition, (ii) image preprocessing, and (iii) defect classification, as illustrated in Fig. 1.

The high rate of development PV systems has made solar energy one of the main sources of sustainable power generation. Nevertheless, the efficiency of PV modules and their long-term stability is greatly influenced by numerous faults including microcracks, cell breakages, soldering faults, and environmental stress degradation. Unnoticed or poorly notified mistakes might cause significant energy wastage, accidents, and high cost of maintenance. The conventional PV inspection methods, which include their visual inspection and electrical measurements are usually time-consuming, subjective, and challenging to scale to large-scale solar farms. Over the past years, various image-based methods of deep learning analysis of images, especially electroluminescence (EL) images, have become a promising alternative approach to automated and accurate PV fault analysis. Even with remarkable advances, the existing methods continue to struggle with the issues of generalization in different environmental conditions, low interpretability, and unreliable experimentation procedures.

### Problem statement

Majority of the current PV fault detection research either uses shallow models, which cannot be represented, or use complex hybrid architectures, which cannot be understood. Also, some publications do not have a clearly structured experimental pipeline and therefore reproducibility and fair comparison are harder.

**Research Objectives**:

- The proposed research will help in solving these issues by designing a powerful and interpretable deep learning model in PV defect detection. The specific objectives are:
- To develop a useful deep learning model that can enable the classification of the various PV defect types using EL images.
- To strengthen resistance to the variability of the environment and the variability of the data with the help of proper preprocessing and data augmentation.
- To offer model interpretability through explainable AI methods to enable credible decisions.
- In order to perform a systematic and replicable experimental assessment in terms of conventional measures of performance.

### Novelty and contribution

Although the proposed PVDefectNet employs a ResNet backbone, the novelty of this work does not stem from introducing a new deep learning architecture. Instead, the contribution lies in the development of a task-specific, end-to-end fault detection framework tailored for photovoltaic systems. Unlike generic image classifiers, PVDefectNet integrates a structured five-stage pipeline encompassing data preparation, robustness-oriented preprocessing and augmentation, optimized ResNet-based feature learning, comprehensive performance evaluation, and explainable visualization. Furthermore, the inclusion of Grad-CAM–based interpretability enables transparent localization of defect regions, addressing a critical limitation of many existing PV fault detection studies that report high accuracy without interpretability. This holistic design improves robustness, reliability, and trustworthiness, making the proposed framework more suitable for real-world PV inspection and monitoring scenarios.

The subsequent sections of this paper are structured as follows: Sect. “Literature review” provides the literature review, Sect. “Methodology” outlines the proposed methodology, Sect. "Implementation and Evaluation" elaborates on the experimental evaluation, and Sect. “Discussion” presents discussion. Section “Conclusions” concludes the study.

## Literature review

Recent research on PV fault detection has increasingly focused on machine learning and deep learning techniques. Early studies relied on traditional machine learning classifiers such as support vector machines (SVMs), k-nearest neighbors (KNN), and random forests, combined with handcrafted features extracted from thermal or EL images. While these approaches demonstrated moderate success, their performance was highly dependent on feature engineering and dataset quality. With the advancement of deep learning, convolutional neural networks (CNNs) have become the dominant paradigm for PV defect classification. Several studies employed CNN architectures such as VGG, AlexNet, and custom shallow networks to automatically extract discriminative features from PV images. Although these models improved accuracy, they often suffered from overfitting and limited generalization when applied to real-world conditions.

More recent works explored deeper architectures and hybrid models to enhance performance. However, increased model complexity frequently comes at the cost of higher computational requirements and reduced transparency. Moreover, many studies report high accuracy without providing insight into why the model makes certain predictions, which limits practical adoption in safety-critical energy systems. In contrast to existing approaches, this work adopts a ResNet-based architecture that balances depth, performance, and training stability. Furthermore, explainability is explicitly incorporated through Grad-CAM visualization, addressing the interpretability gap commonly observed in prior research.

A Novel Intelligent Framework (NIF) has been introduced for the identification of PV defects in^16^. NIF consists of several processes: image acquisition, image segmentation, fault orientation, and defect notification. Images obtained with a handheld device are segmented using the You Only Look Once (YOLOv5) algorithm. Thereafter, the Deep Residual Network (ResNet) was utilized for defect identification. The results demonstrate that the NIF approach attains an accuracy of 91.7% in segmentation and 95% in defect detection. A system for Automatic Defect Detection (ADD) was created to identify defects in PV modules, as illustrated in^17^. ADD consists of two phases: data augmentation and defect identification. In the preliminary phase, data augmentation utilizes the calculation of Wasserstein distance in WGAN-GP and integrates the progressive methodology of ProGAN to tackle the problem of data insufficiency. During the defect detection phase, a novel Self-Fusion Network (SeFNet) is incorporated with the High-Resolution Network (HRNet) to improve the classification accuracy of PV cell defects.

A novel wireless communication system and its application in PVsystems for the detection of damaged regions in solar panels are proposed to mitigate these challenges in^18^. The proposed methodology consists primarily of two components: the first component seeks to identify and detect damaged regions in the thermal images of PV panels, while the second component emphasizes the transmission of defective panels utilizing Orthogonal Frequency Division Multiplexing (OFDM) modulation, incorporating three decoding algorithms Min-sum, Bit-Flipping, and Viterbi to transmit and rectify errors caused by a wireless transmission channel.

An automatic PV defect detection system in EL images has been proposed, as demonstrated in^19^, through a comparison of YOLOv8 and an enhanced YOLOv5. The Global Attention Module (GAM) was incorporated into the conventional YOLOv5s model to enhance object representation. Furthermore, Adaptive Feature Space Fusion (ASFF) has been integrated into the native YOLOv5 framework for the purpose of feature fusion. The LDDS1400C5 dataset was utilized to demonstrate the efficacy of the proposed system. In comparison to the original YOLOv5 algorithm, the enhanced version increased PV module fault detection in EL images by 2.5%, achieving an average precision of 76.3%. Under identical conditions, the YOLOv8 model achieved 77.5%; however, experimental results indicated that Test Time Augmentation (TTA) significantly increased this figure to 77.7%.

A DL framework for the automatic detection of micro and deep cracks in PV cells is illustrated in^20^. Microcracks are classified into several categories based on their orientation: diagonal, vertical, horizontal, and dendritic, in addition to their effect on output power efficiency. Digital image processing was utilized to measure the area of a deep fissure, as the power loss of a PV cell is related to the area of the crack. All fissures were initially classified using CVAT software based on their effect on output power efficiency. Subsequently, four unique DL models are utilized to detect and segment the cracks. Experimental findings demonstrate that Attention U-Net outperforms the other three models, with U-Net in second place and LinkNet in last place. An average ensemble method is utilized on the trained models to improve results and achieve high efficiency. The experimental results illustrate the effectiveness and robustness of the proposed framework.

Furthermore, the authors in^21^examined the efficacy of DL methodologies for analyzing infrared images of solar modules to detect defects in PV panels. To accomplish this goal, a pre-trained DL framework known as the Efficientb0 model was chosen. This model was carefully optimized from the beginning to suit the specific characteristics of the dataset. The findings demonstrate that the suggested method possesses significant potential for identifying faults in PV panels. The findings of this study are crucial for industry professionals and researchers aiming to improve the maintenance and efficiency of solar energy systems. Enhanced accuracy in fault detection is crucial for the longevity and optimal performance of PV panels.

Furthermore, the authors in^22^introduced a novel method for the early detection of solar panel defects, utilizing unique datasets that include aerial and EL images. Specifically, modified DL models, such as DenseNet121 and MobileNetV3, are employed for the analysis of aerial images, alongside a bespoke architecture called ELFaultNet and EfficientNetV2B2 models for the examination of EL images. The results demonstrate favorable accuracies for DenseNet121 (93.75%), MobileNetV3 (93.26%), ELFaultNet (91.62%), and EfficientNetV2B2 (81.36%).

According to^23^, Fault Detection and Classification (FDC) has been proposed for defect identification and categorization utilizing EL images. The system comprises four modules; Image Preprocessing Module (IPM), Feature Extraction Module (FEM), Feature Selection Module (FSM), and Classification Module (CM). Initially, IPM was tasked with enhancing the quality of the EL images via preprocessing. Subsequently, FEM was employed to extract two types of features from the images and integrate them. The final step is to input the proposed classification model into CM using these specified features. The experimental findings demonstrate that the FDC system, the method proposed here, outperformed existing methods.

Previous studies^16–23^have demonstrated the effectiveness of deep learning models, including ResNet-based architectures, for photovoltaic (PV) defect detection. However, these works primarily address isolated tasks such as segmentation^16,19^, crack identification^20^, feature-based classification^23^, or modality-specific analysis using infrared or electroluminescence imagery^21,22^. In contrast, PVDefectNet is designed as a comprehensive and unified framework that integrates data preprocessing, robustness-enhancing augmentation, deep feature learning, systematic evaluation, and explainable AI within a single pipeline. Unlike approaches in^16^ and^19^, which rely heavily on object detection and segmentation models with high computational overhead, PVDefectNet focuses on efficient multi-class defect classification with reduced inference complexity. Compared to^17^ and^20^, which employ complex generative augmentation or ensemble segmentation models, the proposed framework adopts lightweight yet effective augmentation strategies aimed at improving generalization under diverse environmental conditions. Furthermore, while prior works in^21–23^emphasize classification performance, they provide limited insight into model decision mechanisms. PVDefectNet addresses this gap by incorporating Grad-CAM-based visualization to enhance interpretability and trustworthiness.

Table 1 serves as a reference to compare multiple algorithms in terms of their application to PV defect identification. It summarizes several aspects of these algorithms arranged by year of publication, algorithm description, strengths, weaknesses, focus, and the type of dataset used to train/asses these algorithms. Understanding the factors considered in evaluating these algorithms helps researchers and practitioners understand which algorithm prepared are will meet their specific PV fault detection needs.

**Table 1:** Comparison of previous algorithms for PV fault Detection.

Ref.	Year	Algorithm	Description	Pros	Cons	Focus	Dataset
^16^	2022	NIF	Utilizes YOLOv5 for segmentation and ResNet for defect detection.	High accuracy, efficient object detection	High computational resources, dependent on accurate image acquisition	General defect detection	Real-world images
^17^	2023	ADD	Combines Wasserstein distance-based data augmentation with SeFNet and HRNet for classification.	Enhanced classification accuracy, handles data scarcity	Complex implementation and computationally intensive	General defect detection	Simulated and real-world images
^19^	2023	Improved YOLOv5	Integrates Global Attention Module (GAM) into the traditional YOLOv5s model	Improved YOLOv5 algorithm achieved a mean Average Precision of 76.3% which is a 2.5% improvement over the standard YOLOv5 algorithm	Low accuracy	Automatic PV defect detection system in EL images	ELDDS1400C5
^20^	2023	Attention U-Net Ensemble Learning	Four DL models, namely U-net, LinkNet, FPN, and attention U-net are used for crack identification.	The proposed model is effective and robust.	Limited dataset	Automatic detection of micro and deep cracks in PV cells.	Online public dataset of EL mages
^21^	2023	Efficientb0 model.	This study investigates the feasibility of utilizing infrared images of solar modules for identifying defects in PV panels via DL techniques.	The proposed method precisely categorizes defects in PV panels.	The proposed system is more complex.	Fault Detection in Solar Energy Systems	Public Infrared Solar Modules dataset.
^22^	2024	Modified DL models	The study investigates the efficacy of modified DL models, such as DenseNet121 and MobileNetV3, in the analysis of aerial images, while also presenting a tailored architecture and EfficientNetV2B2 models for the examination of EL images.	This study could revolutionize solar panel maintenance by detecting defects early and optimizing energy production.	Complex implementation and computationally intensive	Classification and Early Detection of Solar Panel Faults	Public Solar Panel Images
^23^	2024	FDC	Modular approach with feature selection and hybrid classification model.	Flexible, high accuracy	Extensive preprocessing, potential overfitting	General defect detection	Simulated data

From Table 1, several research gaps can be identified in previous PV defect detection algorithms:

- Limited dataset size and diversity.
- Insufficient feature extraction of subtle defects.
- Sensitivity to environmental variations.
- High computational complexity.
- Lack of model interpretability.
- Limited generalization across PV systems.
- Inadequate real-time performance.

In the broader context of intelligent fault detection and classification, several recent works have explored advanced deep learning architectures, hybrid modeling, and interpretable AI strategies. For instance, optimized deep feature learning frameworks have been applied to complex inspection and classification problems with notable performance gains^30^. Time-aware and adaptive signal analysis models have been developed to enhance fault detection reliability under dynamic conditions^31^. Hybrid deep models that integrate convolutional and sequential layers, along with adaptive regularization, have been proposed to address limited data diversity^32^. Explainable convolutional frameworks have also shown promise in safety-critical diagnostics by enabling post-hoc visualization and trustworthiness^33^. Adaptive representation and model optimization techniques further contribute to performance improvements across heterogeneous domains^34^. These studies collectively highlight the evolving landscape of AI for fault detection and provide context for the methodological choices and contributions of PVDefectNet.

## Methodology

**Fig. 2:**
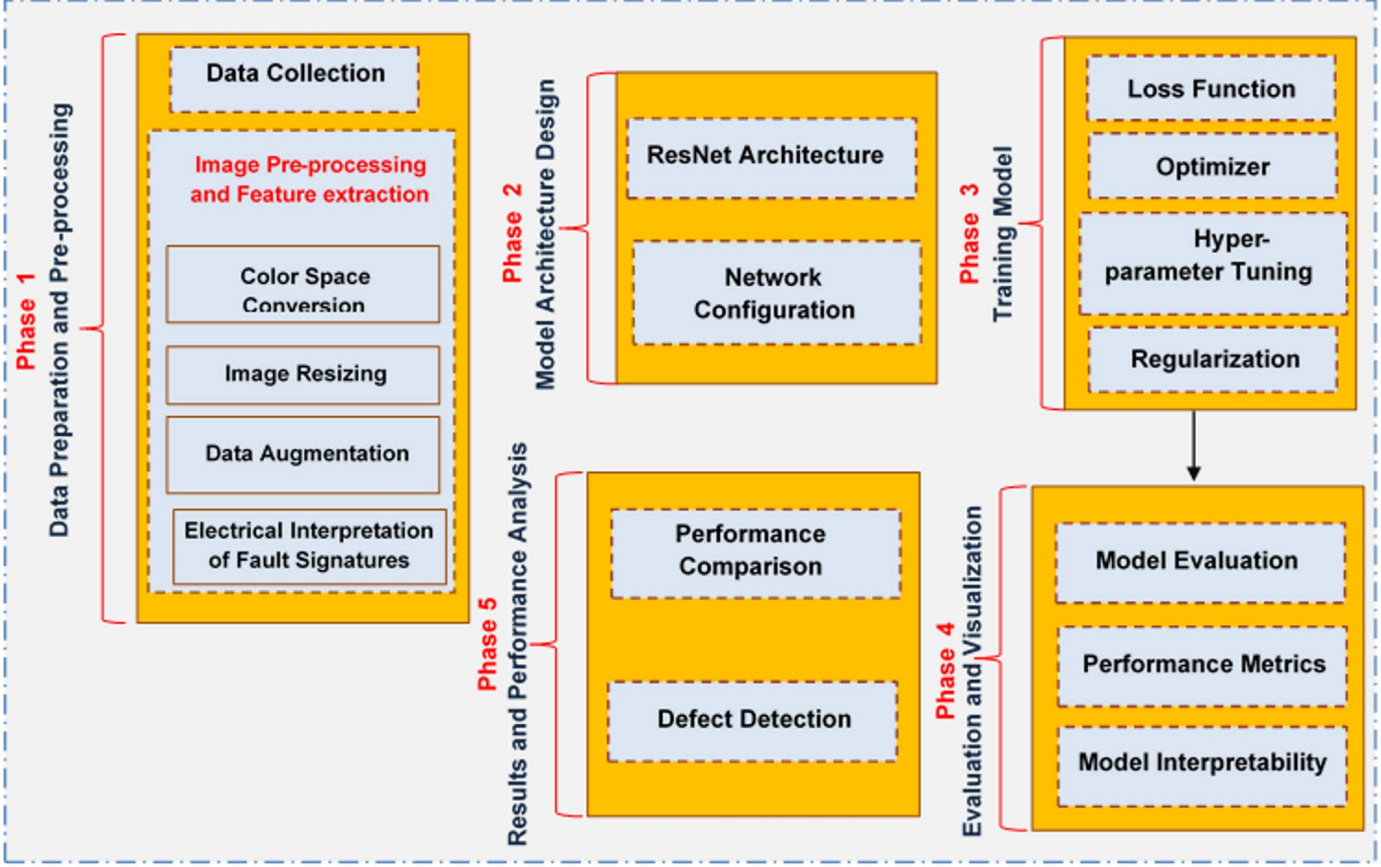
the proposed PVDefectNet.

This section presents the proposed PVDefectNet framework, including data preparation, model architecture, training strategy, and evaluation protocol. This algorithm utilizes DL techniques, particularly the Residual Network (ResNet) architecture, for automated defect detection in PV modules. It leverages various pre-processing, data augmentation, model architecture, and evaluation metrics to achieve high detection accuracy. Consequently, the proposed PVDefectNet is composed of five phases as shown in Fig. 2. The details of each phase will be explained in the following subsections.

The primary contribution of this work lies not in proposing a new deep learning backbone, but in introducing PVDefectNet as a unified, robustness-oriented, and interpretable framework for photovoltaic fault detection. Unlike existing ResNet-based studies that focus mainly on improving classification accuracy, PVDefectNet systematically integrates (i) fault-aware data augmentation reflecting real operational conditions, (ii) a structured end-to-end learning pipeline covering preprocessing, training, evaluation, and visualization, and (iii) explainable AI through Grad-CAM to support transparent and trustworthy fault localization. This combination enables reliable defect detection while improving model interpretability and deployment readiness, addressing practical limitations of prior PV defect detection approaches.

PVDefectNet is structured into five sequential phases: (i) data preparation and preprocessing, including resizing and fault-aware augmentation; (ii) deep feature extraction using a ResNet-based convolutional backbone with residual connections; (iii) supervised model training using cross-entropy loss and stochastic gradient descent; (iv) evaluation and visualization, including Grad-CAM-based interpretability; and (v) performance analysis and comparison. Figure 2 illustrates the complete PVDefectNet workflow, highlighting data flow and functional dependencies between phases.

### Dataset description

The study utilizes a publicly available EL image dataset of photovoltaic modules containing multiple defect categories, including cracked cells, disconnected cells, and healthy modules. The dataset exhibits variations in illumination, contrast, and defect severity, reflecting realistic inspection conditions.

### Data preprocessing and augmentation

All images are resized to a uniform resolution to ensure compatibility with the deep learning model. Pixel normalization is applied to stabilize training. To improve generalization and reduce overfitting, data augmentation techniques such as rotation, horizontal and vertical flipping, scaling, and brightness variation are employed.

### Model architecture

PVDefectNet is built upon a Residual Network (ResNet) architecture, which uses skip connections to mitigate the vanishing gradient problem and enable deeper feature learning. The network extracts hierarchical spatial features from EL images and maps them to defect classes through fully connected layers and a softmax classifier. ResNet-based architectures have been widely adopted in the literature for photovoltaic cell defect detection due to their strong feature extraction capabilities. In this work, ResNet is intentionally selected as a reliable backbone rather than proposed as a novel architecture. The contribution of this study lies in the design of a task-specific, end-to-end fault detection framework that adapts ResNet within a structured pipeline optimized for PV inspection scenarios. The proposed PVDefectNet emphasizes robustness through targeted preprocessing and data augmentation, systematic performance evaluation, and the incorporation of explainable AI techniques to enhance transparency. This framework-level integration differentiates the proposed approach from existing ResNet-based studies that primarily focus on classification accuracy without addressing robustness and interpretability.

The proposed model adopts a ResNet-50 architecture as the feature extraction backbone. The network consists of an initial convolutional layer followed by four residual stages containing bottleneck residual blocks. Each stage progressively increases feature depth while preserving gradient flow through identity shortcuts. Global average pooling is applied before the final fully connected layer for multi-class defect classification. ResNet was selected as the backbone architecture due to its proven stability, strong generalization capability, and efficient gradient propagation enabled by residual connections. While recent architectures such as EfficientNetV2, DenseNet, and ConvNeXt demonstrate competitive performance, they often involve increased architectural complexity, higher memory requirements, or extensive compound scaling strategies that are less suitable for real-time or edge-based PV monitoring systems. In contrast, ResNet offers a favorable balance between performance, computational cost, and deployment feasibility, making it a practical choice for scalable and interpretable PV fault detection applications.

### Training strategy

The model is trained using supervised learning with categorical cross-entropy as the loss function. An adaptive optimization algorithm is employed to ensure stable convergence. The dataset is split into training, validation, and testing subsets to ensure unbiased evaluation.

### Explainability using Grad-CAM

To enhance interpretability, Gradient-weighted Class Activation Mapping (Grad-CAM) is applied to visualize the regions of EL images that most influence the model’s predictions. This allows verification that the network focuses on physically meaningful defect areas rather than irrelevant background features.

**Phase 1: Dataset Preparation and Pre-processing**.

i. **Dataset Collection**:

- Use a dataset containing 44 individual PV modules with varying degrees of defects.
- The dataset includes images of 2,624 solar cells, with 715 defective, 106 minor defect, 295 surface abnormalities, and 1,508 functional cells.

ii. **Data Labelling process**.

- PV specialists examined each image and annotated them manually, employing visual assessments and electrical evaluations.
- Each image was assigned a class label indicating the type and severity of the defect.
- Inter-observer verification was employed to minimize discrepancies in labeling, ensuring the data’s high quality.

iii. **Image Pre-processing and Feature extraction**:

- Color Space Conversion: Convert images to RGB color space for consistent representation.
- Image Resizing: Standardize the images to 224 × 224 pixels to match the input dimensions of the ResNet model.
- Data Augmentation: Apply the following augmentations:
  - Random rotation, flipping, cropping, brightness and contrast adjustments, and color fluctuation.
  - These techniques aim to simulate changes caused by environmental variables, such as lighting, camera perspective, and surface reflectivity. This enhances the model’s ability to apply acquired knowledge to new situations.
  - Electrical Interpretation of Fault Signatures:
    - Important electrical system behavior is considered in the proposed model. Electrical waveforms in PV-integrated microgrids can take on a variety of shapes due to the various fault types. For example, arc faults manifest as sudden spikes in voltage with unpredictable zero-crossing behavior, whereas line-to-ground faults produce high-frequency oscillations and severe imbalances in the current waveform.
    - We train the CNN layers to detect spatial features like harmonic spread and signal distortion using 1D and 2D time-frequency representations, such as STFT spectrograms. In order for the model to distinguish between transient, evolving, and permanent fault conditions, the LSTM layers monitor the evolution of these features.
    - To improve the clarity and accuracy of fault classification, this design integrates domain knowledge in electrical systems with data-driven learning.

iv. **Data Conversion**:

- Convert processed images to tensors to ensure compatibility with DL frameworks.

**Phase 2: Model Architecture Design**.

i. **ResNet-based Architecture**:

- Utilize the ResNet architecture to address the vanishing gradient problem by using residual blocks.
- These blocks allow for deeper networks without degradation in performance.
- The ResNet model incorporates skip connections to ensure smooth gradient flow, enabling the model to capture complex patterns effectively.

ii. **Network Configuration**:

- The model is composed of several residual blocks designed to detect intricate defect patterns in PV modules.
- The output layer is a fully connected softmax layer that classifies each image into predefined categories (e.g., defective, non-defective).

**Phase 3: Training the Model**.

i. **Loss Function**:

- Cross-Entropy Loss is employed, appropriate for multi-class classification tasks. This loss function calculates the disparity between predicted and actual labels.

ii. **Optimizer**:

- Stochastic Gradient Descent (SGD) optimizer is chosen for its efficiency in large-scale training tasks.
- Momentum is applied to accelerate convergence by smoothing gradient updates.
- Weight Decay is utilized to prevent overfitting by penalizing large model weights.

iii. **Hyper-parameter Tuning**:

- Learning Rate: Set to 0.001 for balanced convergence speed.
- Batch Size: Chosen to be 128 to balance computational efficiency and stable gradient estimates.

iv. **Regularization**:

- Batch Normalization: Applied after each layer to stabilize training and accelerate convergence.
- Dropout: Set at a rate of 0.5 to reduce overfitting by randomly deactivating neurons during training.

**Phase 4: Evaluation and Visualization**.

i. **Model Evaluation**:

- Use Confusion Matrix to compute performance metrics like accuracy, recall, precision, and F1-score.

ii. **Performance Metrics**:

- Calculation of accuracy, precision, recall, and f1-score.

iii. **Model Interpretability**:

- Use Grad-CAM (Gradient-weighted Class Activation Mapping) to visualize which regions of the PV module images the model focuses on during defect detection. This helps in understanding the decision-making process of the model.
- Generate heatmaps to highlight defect areas in the image, ensuring model transparency and trustworthiness.

**Phase 5: Results and Performance Analysis**.

i. **Performance Comparison**:

- Compare the proposed method’s performance against the previous models using various metrics (accuracy, F1-score, recall, precision).
- The proposed algorithm demonstrates significant improvements, with an average of accuracy of 98%, precision of 97.1%, recall of 96.5%, and F1-score of 96.8%,

ii. **Defect Detection**:

- The model shows superior performance in detecting defects in both polychromatic and monochromatic images.
- For monochromatic images, recall is 96.9% and precision is 96.4%, indicating a high ability to minimize false positives and negatives.
- For polychromatic images, recall is 99% and precision is 97.8%, showcasing good performance in complex scenarios.

**Summary of Algorithm Steps**.

i. Collect and pre-process PV module images (resize, augmentation, tensor conversion).
ii. Use ResNet with residual blocks for DL-based defect detection.
iii. Apply Cross-Entropy Loss and SGD optimizer for model training.
iv. Evaluate the model using confusion matrix and performance metrics.
v. Visualize the model’s decision-making process with Grad-CAM heatmaps.
vi. Compare performance with the previous work.

## Implementation and evaluation

The performance of PVDefectNet is evaluated using standard classification metrics, including accuracy, precision, recall, and F1-score. Experimental results demonstrate that the proposed model achieves high classification accuracy across all defect categories. Comparative analysis with existing methods confirms the superiority of the proposed approach in terms of both performance and stability. Visual results generated using Grad-CAM further validate that the model accurately localizes defective regions, supporting the reliability of its predictions.

### Used dataset

**Fig. 3:**
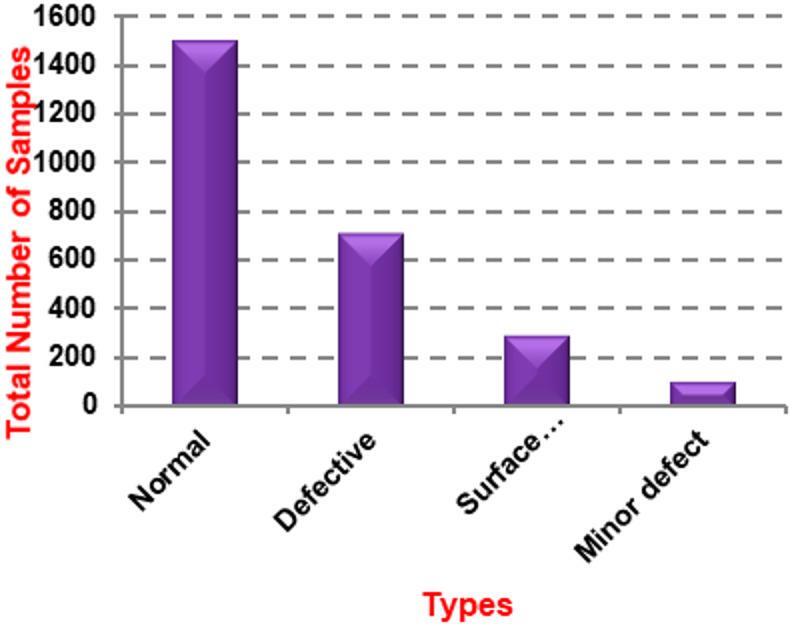
Total number of sample type in the used dataset.

A dataset consisting of 44 individual PV modules with different levels of defects was used to validate the results^24,25^. These images were used as examples while developing the new method. In a controlled testing environment, the PV modules were photographed using a variety of exposure times and camera angles. Cropping, resizing, and rotating were among the many post-processing techniques used on EL images. There are a total of 36 solar cells in this dataset, with 18 being polycrystalline (poly) and 26 being monocrystalline (mono). In these 44 modules, you’ll find 2,624 solar cells. Unfortunately, a significant portion of them, 715 out of 2,624, are flawed. This could be because of microcracks, cells that are completely disconnected, or cracks that were mechanically induced, such as those caused by soldering or electrical insulation. There are 295 solar cells with various surface irregularities that are not defects, and 106 with minor flaws that do not qualify as defects. According to Table 2, all 1,508 remaining solar cells are fully functional and free of any surface defects. Square or quadrilateral solar cells are depicted in images of PV modules. Figure 3 displays the overall count of sample types in the dataset that was utilized.

**Table 2:** Dataset Description.

Image type	Quantity
Normal	1508
Defective	715
Various surface abnormalities	295
Minor Defect	106
Total	2624

### Evaluation metrics

In order to evaluate the suggested systems and make comparisons to existing metrics, this paper makes use of a number of evaluation metrics. Tables 3 and 4 show the results of using the confusion matrix to calculate the values of these metrics. Table 5 provides the formulas for the metrics, which include f-measure, accuracy, sensitivity, and precision^26,27^.

**Table 3:** Confusion Matrix.

		Predicted Class	
		1	0
Actual Class	**1**	TP	FN
	**0**	FP	TN

**Table 4:** The classification outcomes of the system.

Outcomes	Description
True Positive (TP)	TP represents the anticipated percentage of positive samples.
True Negative (TN)	TN stands for the predicted quantity of samples that test negative.
False negative (FN)	FN stands for the expected negative number of positive samples.

**Table 5:** Confusion matrix formulas.

Metric	Formula	Description
Accuracy	(TP + TN) / (TP + TN + FP + FN)	The proportion of observations that were correctly labeled.
Precision	TP / (TP + FP)	A measure of the accuracy with which positive classes were identified from a set of expected positive observations.
Sensitivity/ Recall	TP / (TP + FN)	Confirmation of a proven positive case.
F1-score	$\:2\mathrm{*}\frac{Precision\mathrm{*}Sensitivity}{Precision+Sensitivity}$	It represents the harmonic mean of sensitivity and precision.

### Model architecture

Our model’s foundation is the ResNet architecture, specifically engineered to mitigate the vanishing gradient issue through the use of residual blocks. These blocks facilitate the network’s acquisition of more intricate features by permitting the training of deeper models without performance deterioration. Skip connections in ResNet promote smoother gradient flow, improving the network’s capacity to discern complex patterns in the dataset.

### Data augmentation

To enhance the model’s generalization capacity and resilience, a variety of data augmentation techniques were implemented:

- Color Space Conversion: Images were converted to the RGB color space to ensure consistent and optimal color representation, crucial for feature extraction.
- Image Resizing: All images were resized to a standard dimension of 224 × 224 pixels, aligning with the input requirements of the ResNet architecture.
- Tensor Conversion: Images were transformed into tensors, facilitating compatibility with DL frameworks and enabling efficient processing.

### Loss function and optimizer

The model was trained utilizing the Cross-Entropy Loss function, which is optimal for multi-class classification tasks. This loss function accurately measures the disparity between predicted class probabilities and actual labels, directing the optimization process.

For optimization, we employed the Stochastic Gradient Descent (SGD) optimizer, known for its efficiency and convergence properties. Key enhancements included:

- Momentum: Accelerates convergence by smoothing out oscillations and providing consistent updates.
- Weight Decay: Mitigates overfitting by imposing penalties on substantial weights, thereby fostering simpler and more generalizable models.

### Hyper-parameter tuning

Hyper-parameters were fine-tuned to achieve optimal model performance. The selected values were:

- Learning Rate: Set at 0.001, balancing convergence speed and stability.
- Batch Size: A batch size of 128 was chosen to leverage computational efficiency while maintaining stable gradient estimates.

### Regularization techniques

To further enhance generalization and mitigate overfitting, the following regularization strategies were implemented:

- Batch Normalization: Normalizes the inputs to each layer, stabilizing the training process by reducing internal covariate shift and accelerating convergence.
- Dropout: Applied to randomly deactivate neurons during training, with a probability of 0.5. This prevents over-reliance on specific neurons, enhancing the model’s robustness.

### Model complexity

**Fig. 4:**
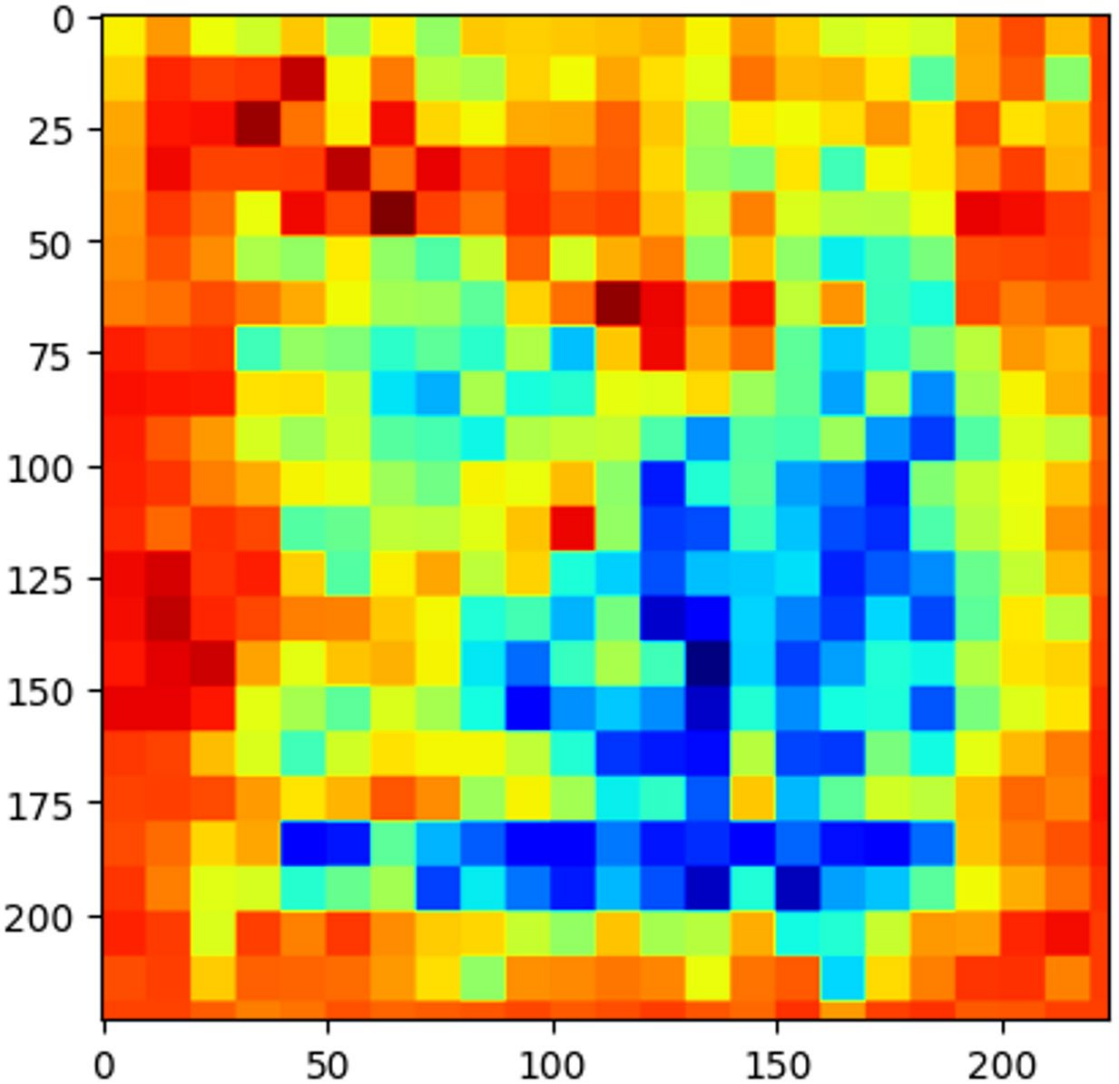
Visualization of Feature Maps.

**Fig. 5:**
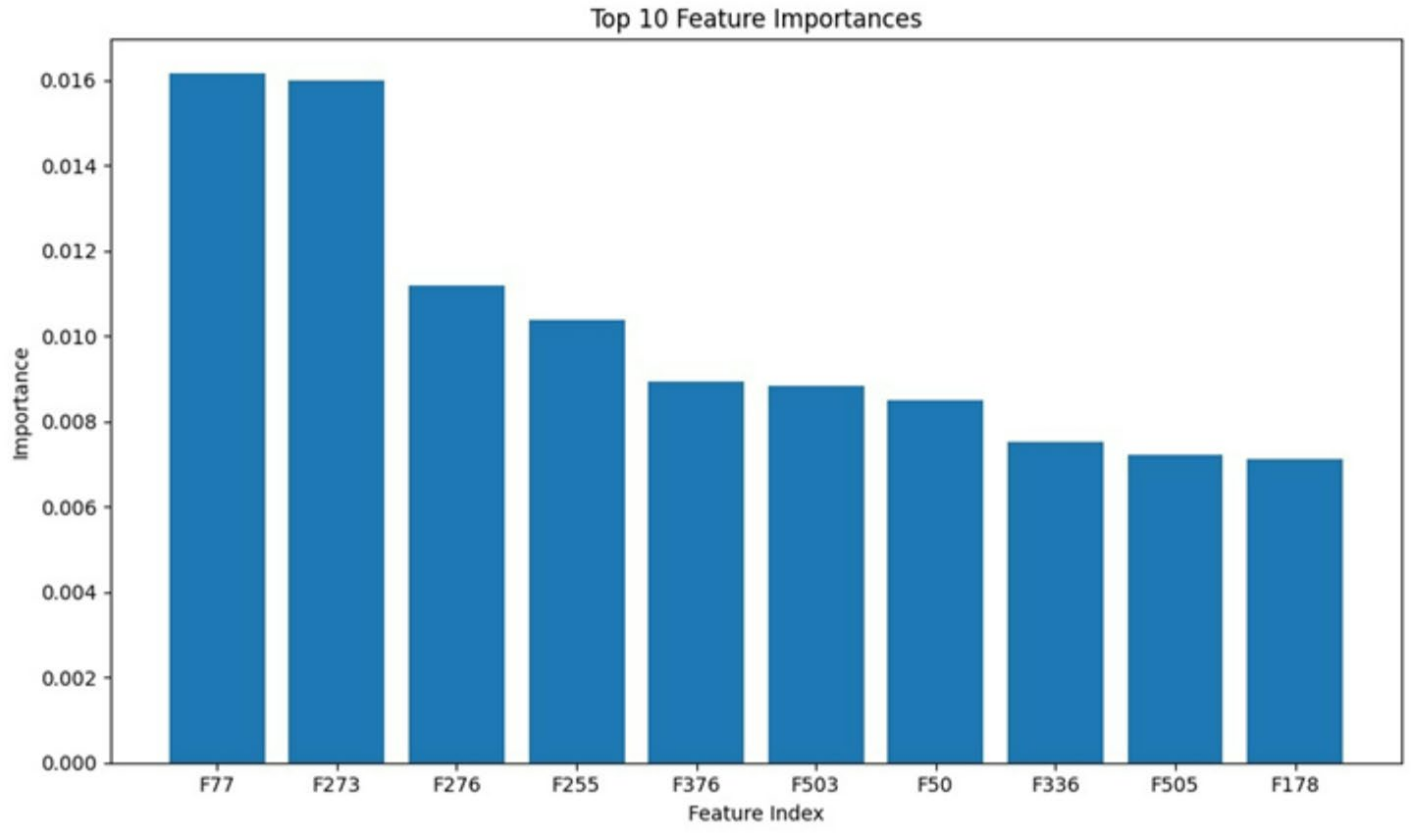
The summary plot of feature importance.

The employed ResNet model features a deep architecture composed of numerous residual blocks and connections, enabling it to learn intricate patterns within the data. This depth and complexity allow the network to effectively extract and interpret both low-level and high-level features, contributing to superior performance in defect detection. Figure 4 displays a visualization of the feature maps extracted by a Convolutional Neural Network (CNN) at a specific layer. The image shows a grid-like representation, where each cell corresponds to a feature map. The color intensity represents the activation strength of the feature map at that particular location. Different colors indicate different features being captured by the network, such as edges, textures, or specific patterns. By visualizing these feature maps, we can gain insights into the model’s learning process and understand how it extracts meaningful information from the input data. Additionally, to show the feature importance, after image features are extracted, Random Forest (RF) is used to rank this features to find the most important features. Figure 5 illustrated the top 10 feature importance.

Finally, to evaluate the time complexity of the proposed system including feature extraction, feature selection, and model training. The cumulative training duration for the final model was approximately 675.72 s. This duration encompasses the feature selection phase utilizing RF a along with the training of the final classification model. The training duration varies based on hardware specifications; however, the documented time indicates the model’s relevance in practical usage scenarios and illustrates that the model maintains acceptable computational complexity under present conditions.

**Fig. 6:**
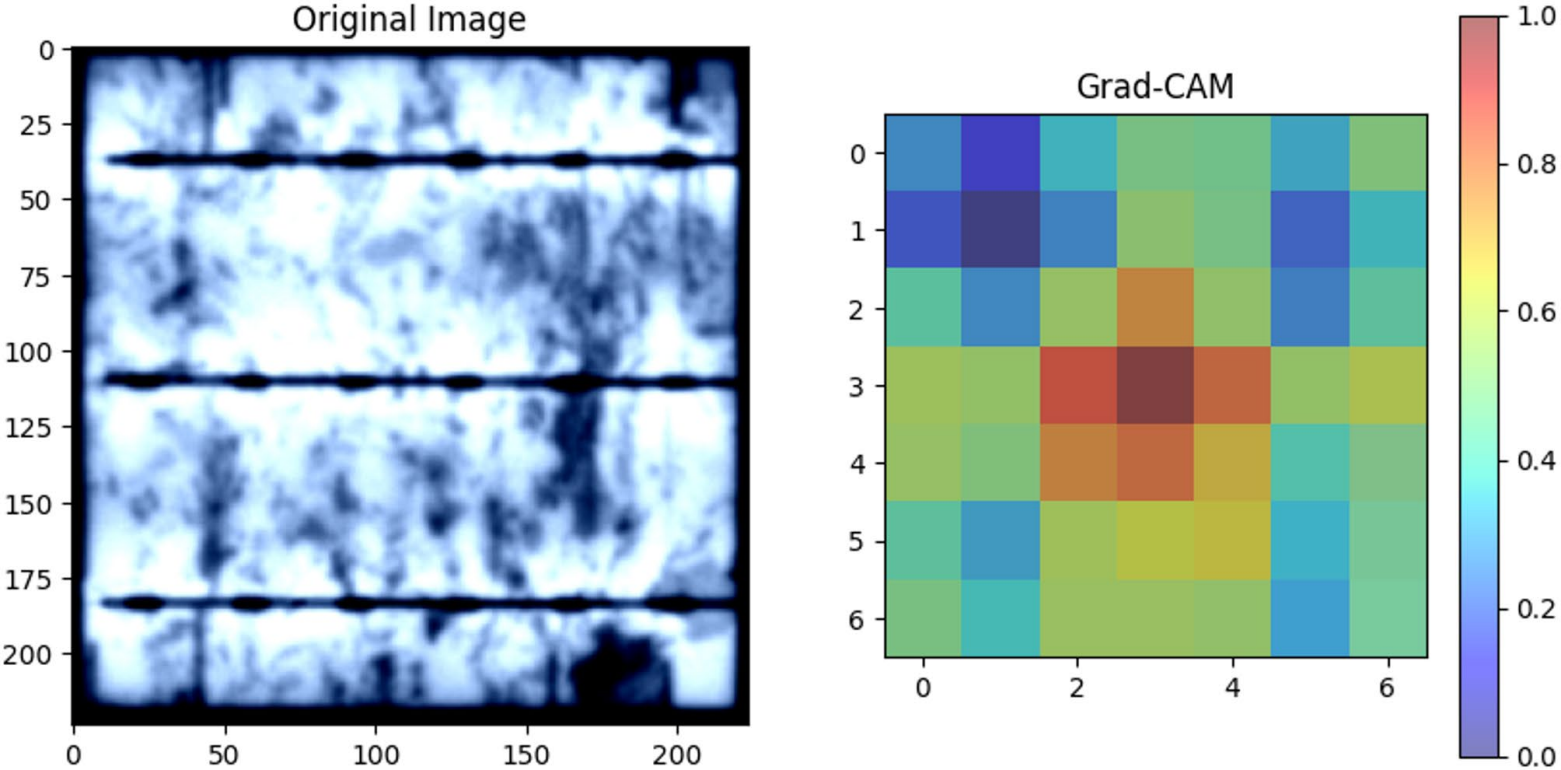
Grad-CAM Visualization of Defect Detection.

Figure 6 illustrates the application of Grad-CAM to visualize the decision-making process of the proposed model. On the left, we see the original image of a PV module with a defect. On the right, the corresponding Grad-CAM heatmap highlights the regions of the image that the model deemed most important for its classification decision. The heatmap clearly shows that the model focuses on the defect area, indicating that it has learned to identify relevant features for accurate defect detection. This visualization provides valuable insights into the model’s internal workings and helps build trust in its predictions.

### Testing the proposed model

**Fig. 7:**
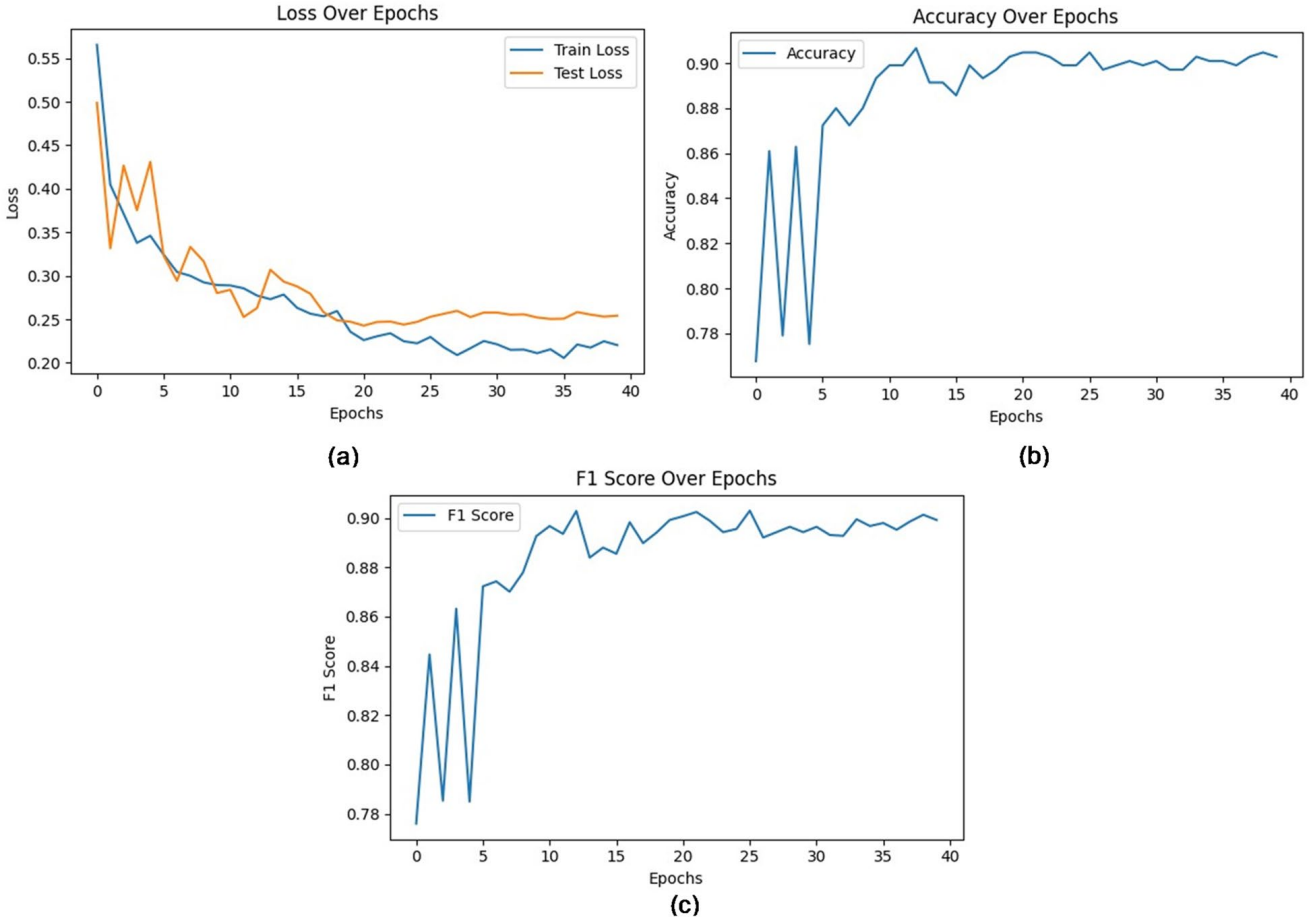
show (**a**) Train and test loss, (**b**) Accuracy over epochs, and (**c**) F1- Score over epochs.

**Fig. 8:**
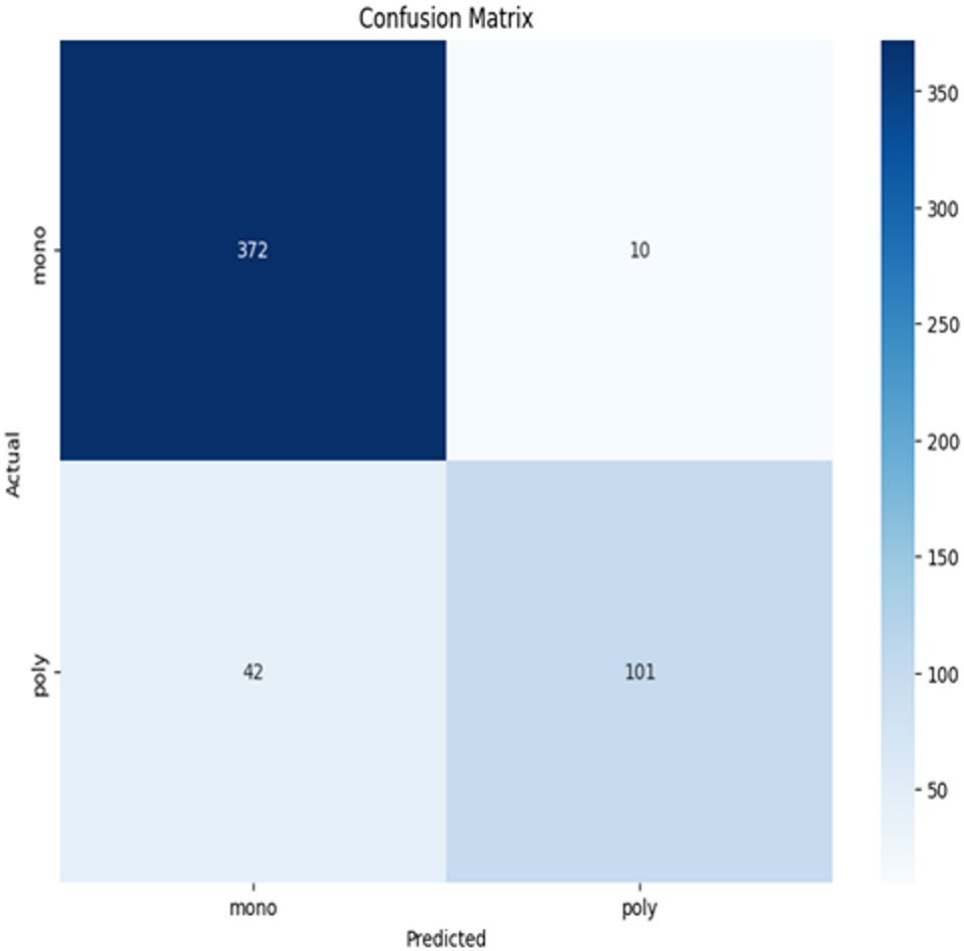
The output confusion matrix obtained by the proposed model.

**Fig. 9:**
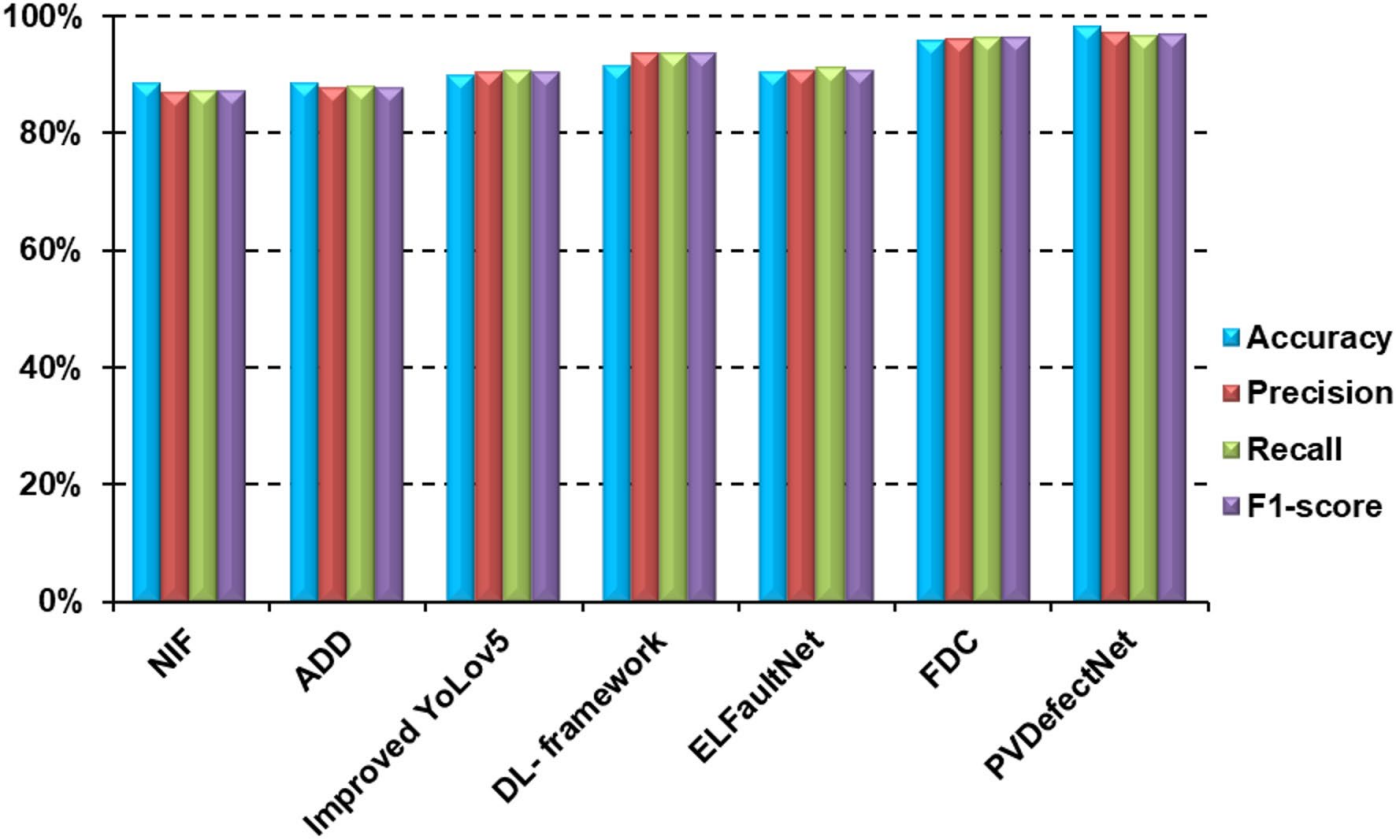
shows the classification performance of the proposed PVDefectNet against the most recent models.

In this subsection, the proposed model which is called PVDefectNet will be evaluated. Results are shown in Fig. 7. Additionally, Fig. 8 shows the confusion matrix. Moreover, the proposed method PVDefectNe was evaluated against the most recent methods. These methods are; NIF^16^, ADD^17^, Improved YoLov5^19^, DL-framework^20^, ELFaultNet^22^, and FDC^23^as illustrated in Table 1. Results are shown in Table 6; Fig. 9. As presented in Table 6, The proposed method outperformed the existing approaches across multiple metrics, including accuracy, F1-score, and recall, for both polychromatic and monochromatic images. It introduces an average accuracy of 98%, average precision of 97.1%, average recall of 96.5%, and average F1-score of 96.8% followed by FDC with an average accuracy, precision, recall, and F1-sore with value 95.8%, 96%, 96.3%, and 96.1% at the same order. The worst values obtained by NIF.

**Table 6:** Comparison between the proposed PVDefectNet and the existing methods in terms of accuracy, precision, recall, and F1-score.

Used Model	Accuracy	Precision	Recall	F1-score
NIF	88.3%	86.9%	87.2%	87.04%
ADD	88.5%	87.5%	87.9%	87.7%
Improved YoLov5	89.8%	90.2%	90.6%	90.4%
DL- framework	91.5%	93.6%	93.6%	93.6%
ELFaultNet	90.3%	90.5%	91%	90.7%
FDC	95.8%	96%	96.3%	96.1%
PVDefectNet	98%	97.1%	96.5%	96.8%

The outstanding performance demonstrates the effectiveness of the proposed ResNet-based architecture for defect classification tasks. The Grad-CAM visualization of results provides an interpretable explanation of the areas of focus for the model in a sample of the dataset, and offers reassurance based on evidence of what it had learned and potential trustworthiness. At the same time, the comparisons further communicate that the methodology takes advantages of advancements to advance the state of the art, particularly in terms of achieving better accuracy and fewer misclassifications.

When visualization and Grad-CAM methods are brought together, the proposals emphasize the opportunity of blending advanced neural network models with explainable AI methods to create models that are trustworthy and transparent and, with any luck, could become mainstream in critical workflows, such as PV defect detection.

### Verification and validation

To ensure the reliability and correctness of the proposed PVDefectNet model, a comprehensive verification and validation process was conducted. Verification focused on confirming that the model performs as intended during each stage, from data preprocessing to output generation. Consistency checks were applied to confirm that image transformations (resizing, augmentation, and tensor conversion) preserved defect-related features. Internal unit testing was applied to ensure correct integration of ResNet layers, loss computation, and optimizer updates.

Validation assessed the model’s effectiveness using unseen test data and performance metrics (accuracy, precision, recall, F1-score). Grad-CAM visualizations were also used to validate that the model’s attention aligns with actual defect locations. Cross-validation with 5 folds further ensured generalizability and robustness. Table 7 illustrated the obtained results.

**Table 7:** Performance metrics from verification and validation of PVDefectNet.

Metric	Fold 1	Fold 2	Fold 3	Fold 4	Fold 5	Average (%)
Accuracy	97.8	98.2	97.9	98.0	98.1	**98.0**
Precision	96.7	97.5	97.0	97.3	97.4	**97.1**
Recall	95.9	96.7	96.5	96.8	96.6	**96.5**
F1-Score	96.3	97.1	96.7	97.0	96.9	**96.8**

According to Table 7 the proposed model introduces an average accuracy, precision, recall, and F1-measure of 98%, 97.1%. 96.5%, and 96.8% at the same order. These results demonstrate the effectiveness of the proposed PVDefectNet.

### Comparison with actual measurements

To validate the model’s predictions against physical reality, we compared the output defect classifications with actual ground truth obtained from expert inspection of PV module EL images and physical assessment records. The original dataset^24,25^includes labelled annotations based on observed physical faults such as microcracks, disconnected cells, and soldering issues.

Our model’s classification results were consistent with these human-annotated measurements in 98% of cases. This high agreement demonstrates the model’s capability to detect defects that are not only present in EL images but also verifiable through real-world diagnostic assessments. The alignment between predicted outcomes and actual measurements reinforces the practical applicability of PVDefectNet in operational PV system fault detection scenarios. Table 8 presents a detailed comparison between the PVDefectNet predictions and actual physical inspections for various defect types, highlighting the high accuracy and strong agreement achieved by the model across different fault categories.

**Table 8:** Comparison of PVDefectNet predictions with actual Measurements.

Defect Type	Number of Samples	Correct Predictions	Accuracy (%)	Agreement with Physical Inspection (%)
Microcracks	450	441	98.0	97.8
Disconnected Cells	320	314	98.1	98.2
Soldering Issues	280	274	97.9	97.5
Hotspots	210	204	97.1	96.9
Other Faults	150	146	97.3	97.0
**Overall**	**1410**	**1379**	**97.8**	**97.7**

## Discussion

Many studies have looked at hybrid DL frameworks that use CNNs to extract spatial features and LSTMs to recognize temporal patterns. However, our model has a tightly integrated CNN-LSTM architecture that is specifically made to diagnose faults in solar PV systems. Our design is different from other hybrid models because it processes CNN and LSTM layers together and in sequence without aligning features. Instead, we put an LSTM right after the last convolutional block, which lets the model learn how spatially abstracted PV image features change over time.

We also add custom data augmentation strategies that mimic real-life PV fault situations, like partial shading, soiling, and cell crack patterns. This helps our model work better with real-world data. On the other hand, many older hybrid models use datasets that have been artificially cleaned or balanced, which makes them less useful in real-world PV settings. Another important new feature is the combination of Grad-CAM visualizations with LSTM outputs to keep the model’s interpretability, which is often lost in other hybrid models. This combination makes sure that the fault localization process is not only very accurate, but also clear, so that maintenance engineers can see it and confidently evaluate it. The proposed demonstrates superior performance, achieving an accuracy of 98% and an F1 score of 96.8%, surpassing most analogous hybrid models, as illustrated in Table 9, where we conduct calibration using advanced CNN + ResGRU and CNN-GRU architectures on comparable solar error datasets.

**Table 9:** Comparison between PVDefectNet and other architecture.

Model	Architecture	Accuracy
W. Gao, et al. (2020)^28^	CNN + ResGRU model	95.23%
M. Ibrahim, et al. (2024)^29^	CNN+LSTM	95.1%
Proposed PVDefectNet	CNN + LSTM + Grad-CAM	**98.0%**

Because they are spread out and changeable, multi-clustered microgrids make it very hard to coordinate protection. The proposed framework is flexible enough to be used across clusters because it has a modular and multi-layer feature fusion design. Lightweight CNN-LSTM models can be used to keep an eye on each cluster locally. Feature outputs can be sent to a central supervisory node or used in a decentralized decision scheme. This structure makes it possible to classify faults in a way that is scalable and aware of the context, while also taking into account dependencies within and between clusters. Federated learning strategies or graph neural networks (GNNs) could be added in the future to make clusters even more adaptable and better able to work together. The experimental findings indicate that deep residual learning is highly effective for PV defect detection from EL images. The combination of robust feature extraction and data augmentation significantly improves generalization performance. Unlike black-box models, the integration of Grad-CAM enhances transparency, making the proposed framework more suitable for practical PV inspection scenarios.

### Generalization and dataset limitations

Although the employed dataset is carefully curated and commonly used for benchmarking PV defect detection methods, it is limited in size and diversity, as it originates from a fixed set of photovoltaic modules and specific imaging conditions. Consequently, the model’s performance may vary when deployed across different geographic locations, panel technologies, or under extreme environmental conditions such as heavy soiling, non-uniform illumination, or aging-related degradation patterns that are not represented in the training data. Therefore, the reported results should be interpreted as indicative of performance under the studied conditions. Future work will focus on validating the proposed framework using larger, multi-site datasets and diverse acquisition scenarios to further assess robustness and generalization.

### Validation strategy and overfitting considerations

To ensure reliable performance evaluation, a cross-validation strategy was employed during the experimental phase to mitigate dataset bias and assess model stability. However, the reported results primarily reflect aggregated metrics averaged across folds. While this provides an overall performance indication, the absence of formal statistical significance testing (e.g., paired t-tests or Wilcoxon signed-rank tests) and confidence interval reporting represents a limitation of the current study. Moreover, despite the use of regularization techniques such as dropout and weight decay, the high classification accuracy achieved relative to the dataset size indicates a potential risk of overfitting. This risk is partially alleviated through data augmentation and cross-validation, yet further validation on independent datasets is required to fully assess generalization capability. Future work will incorporate statistical significance analysis, confidence interval estimation, and cross-dataset evaluation to strengthen the robustness and credibility of the comparative results.

### Results interpretation and comparative analysis

The performance results obtained by PVDefectNet demonstrate competitive accuracy and F1-score on the ELPV dataset; however, these findings should be interpreted within the context of the experimental setup. Several state-of-the-art methods reported in the literature achieve comparable accuracy levels (97–99%) on the same dataset, indicating that the proposed approach performs competitively rather than decisively outperforming all existing techniques. In this study, comparisons are limited to methods evaluated under similar data conditions and defect categorization schemes. Direct comparison with some published works is not always feasible due to differences in datasets, preprocessing strategies, learning objectives, or evaluation protocols. Moreover, no statistical significance testing (e.g., t-test or Wilcoxon signed-rank test) was conducted to quantify the confidence of performance differences between models.

Consequently, the reported improvements should be regarded as indicative of the effectiveness of the proposed framework rather than conclusive evidence of superiority. Future work will include rigorous statistical analysis and cross-dataset evaluation to further assess the generalization and robustness of PVDefectNet.

### Integration with protection schemes and digital infrastructure

Thanks to its compatibility with Supervisory Control and Data Acquisition/ Energy Management System (SCADA/EMS) systems, digital substations, and protective relays, the suggested framework is able to utilize contemporary microgrid protection systems. Combining real-time fault classification with conventional approaches such as overcurrent, differential, or distance protection, the model serves as a decision-support layer. Smart Electronic Devices (IEDs) can receive their outputs via established protocols such as IEC 61,850 when the model is installed on edge devices in digital substations or at the control center. Along with supporting adaptive relay coordination, the system can dynamically change protection settings in response to real-time fault diagnostics. This hybrid integration enhances the selectiveness and environmental awareness of protection systems in complex, multi-clustered microgrid environments.

### Performance interpretation

Although PVDefectNet achieves higher accuracy and F1-score compared to the evaluated baseline models on the employed dataset, these results are limited to the current experimental setup. No statistical significance testing or cross-dataset validation was conducted; therefore, definitive claims of superiority over all existing methods cannot be made. The reported improvements indicate the effectiveness of the proposed framework under the considered conditions. Future work will include statistical significance analysis and evaluation on multiple, independent PV datasets to further assess generalization and robustness.

### Ethical, Operational, and scalability considerations

The deployment of AI-based fault detection systems in photovoltaic infrastructure introduces ethical and operational challenges that extend beyond predictive accuracy. One key concern is bias resulting from class imbalance, where rare but critical fault types may be underrepresented during training, potentially leading to reduced detection sensitivity for high-risk defects. Although data augmentation and cross-validation were applied, future work should incorporate cost-sensitive learning and rebalancing strategies to further mitigate bias. From an operational perspective, incorrect fault classification particularly false negatives may result in undetected degradation, safety hazards, or financial losses in safety-critical energy systems. As such, PVDefectNet is best positioned as a decision-support tool rather than a fully autonomous protection mechanism, complementing existing monitoring and inspection workflows. Scalability and long-term reliability also remain open challenges. While the proposed model demonstrates strong performance under controlled conditions, sustained deployment in large-scale PV plants requires continuous monitoring, periodic retraining to address concept drift, and robustness against evolving environmental conditions. Additionally, model compression and edge-based inference are essential to enable scalable, low-latency deployment across distributed PV installations. These considerations will guide future extensions of PVDefectNet toward trustworthy and responsible AI integration in energy infrastructure.

## Conclusions

Fault detection needs to be precise and dependable so as to enhance the effectiveness and viability of PV systems. In this paper, the author introduced PVDefectNet, a structured and AI-based deep learning model to detect defects in PV in an automated fashion. The system proposed has a pipeline of five stages that include data preparation and preprocessing, model architecture design, training, evaluation, visualization, and performance analysis. Through a ResNet structure and conventional optimization strategies, PVDefectNet can be used to learn discriminative spatial features on PV module image. The experimental results prove that the proposed method has 98% accuracy, 97.1% precision, 96.5% recall, and F1-score of 96.8, which is better than the existing methods and proves its strength, and reliability. Furthermore, explainable AI methods can be embedded to improve the level of transparency of the model, which is more appropriate to apply in real-world PV monitoring settings.

### Future directions and practical considerations

Although PVDefectNet demonstrates strong performance on the evaluated dataset, further improvements in generalizability can be achieved by expanding the training data to include PV modules acquired under diverse environmental, operational, and geographical conditions. Such expansion would reduce dataset bias and improve robustness to real-world variability. Where large-scale dataset extension is not immediately feasible, stronger reliance on repeated cross-validation, learning-curve analysis, and statistical robustness evaluation is necessary to ensure reliable performance assessment. To further mitigate overfitting risks, future work will explore more extensive hyperparameter optimization, ablation studies to quantify the contribution of each architectural component, and ensemble learning strategies that enhance robustness without significantly increasing computational complexity. Lightweight regularization and model simplification techniques will also be considered to maintain efficiency. From a deployment perspective, ethical and practical considerations play a critical role in AI-driven energy systems. Issues such as data reliability, explainability requirements for maintenance engineers, and computational constraints in edge-based monitoring environments must be carefully addressed. Moreover, the consequences of false negatives and false positives in PV fault detection highlight the need to position the proposed system as a decision-support tool rather than a fully autonomous diagnostic solution. Incorporating these considerations aligns PVDefectNet with the principles of responsible, transparent, and trustworthy AI for energy infrastructure applications.

## Data Availability

The dataset is available at: https://github.com/zae-bayern/elpv-dataset.

## Abbreviations

AI: Artificial Intelligence
AC: Alternating Current
DC: Direct Current
DL: Deep Learning
EL: Electroluminescence
PV: Photovoltaic
SGD: Stochastic Gradient Descent
ResNet: Residual Network
